# Deciphering the Tissue Tropism of the RNA Viromes Harbored by Field-Collected Anopheles sinensis and Culex quinquefasciatus

**DOI:** 10.1128/spectrum.01344-22

**Published:** 2022-08-15

**Authors:** Yan-Jun Kang, Guoding Zhu, Yang Cheng, Haitao Yang, Jun Cao

**Affiliations:** a Department of Pediatric Laboratory, Wuxi Children's Hospital, Wuxi, China; b National Health Commission Key Laboratory of Parasitic Disease Control and Prevention, Jiangsu Provincial Key Laboratory on Parasite and Vector Control Technology, Jiangsu Institute of Parasitic Diseases, Wuxi, China; c Wuxi School of Medicine & Public Health Research Center, Jiangnan University, Wuxi, China; d Center for Global Health, School of Public Health, Nanjing Medical University, Nanjing, China; e Jiangsu Health Development Research Center, Nanjing, China; Hubei University of Medicine

**Keywords:** insect-specific viruses, virome, tissue tropism, *Anopheles sinensis*, *Culex quinquefasciatus*

## Abstract

Arboviruses and insect-specific viruses (ISVs) are two major types of viruses harbored by mosquitoes that are distinguished by the involvement of vertebrate hosts in their transmission cycles. While intensive studies have focused on the transmission, tissue tropism, and evolution of arboviruses, these characteristics are poorly investigated in ISVs, which dominate the mosquito virome. Therefore, in this study, we collected two mosquito species, Anopheles sinensis and Culex quinquefasciatus, in the field and used a metatranscriptomics approach to characterize their RNA viromes in different tissues, such as the midgut, legs, salivary gland, eggs, and the remainder of the carcass. Blood-engorged individuals of these species were captured in 3 locations, and 60 mosquitoes were pooled from each species and location. A total of 40 viral species from diverse viral taxa associated with all viral RNA genome types were identified, among which 19 were newly identified in this study. According to the current viral taxonomy, some of these viruses, such as Yancheng *Anopheles* associated virus 2 (*Narnaviridae*) and Jiangsu *Anopheles*-related virus (*Ghabrivirales*), were novel. The two investigated mosquito species generally harbored distinct viromes. Nevertheless, the viruses were generally shared among different tissue types to various degrees. Specifically, the eggs possessed a viral community with significantly lower diversity and abundance than those in other tissues, whereas the legs and salivary glands exhibited higher viral abundance. The compositions and distributions of the viromes of different mosquito tissues were demonstrated for the first time in our study, providing important insight into the virome dynamics within individual mosquitoes.

**IMPORTANCE** ISVs are considered to be ancestral to arboviruses. Because of their medical importance, arboviruses have been well studied from the aspects of their transmission mode, evolution of dual-host tropism, and genetic dynamics within mosquito vectors. However, the mode of ISV maintenance is poorly understood, even though many novel ISVs have been identified with the emergence of sequencing technology. In our study, in addition to the identification of a diverse virus community, the tissue tropism of RNA viromes harbored by two field-collected mosquito species was demonstrated for the first time. According to the results, the virus communities of different tissues, such as the salivary glands, midguts, legs, and eggs, can help us understand the evolution, transmission routes, and maintenance modes of mosquito-specific viruses in nature.

## INTRODUCTION

As the most diversified lineage within the kingdom Animalia, class Insecta is perceived as a reservoir of viruses with great diversity (1). According to the virus transmission mode, the viruses associated with insects can be divided into two major groups: arthropod-borne viruses (arboviruses) and insect-specific viruses (ISVs) (2). The transmission of arboviruses occurs when hematophagous arthropod vectors, such as mosquitoes or ticks, bite a susceptible vertebrate host with viremia. Then, arboviruses undergo replication and transmission within the insect to cause a systemic infection (3). Effective arboviral circulation within the female mosquito body begins in the midgut containing the blood meal from the host with viremia, where the viruses to which the midgut epithelium is susceptible multiply productively and then disseminate into the hemocoel. With the aid of hemolymph circulation, if viral replication in the salivary gland is possible, horizontal transmission can eventually proceed (4). During this process, the tropism of the viruses toward tissue cells greatly impacts their transmission and maintenance in nature. In addition, innate immune pathways, such as the antiviral RNA interference pathway within mosquito vectors, can affect the transmission efficiency of viruses (5). Therefore, both tissue tropism and vector immune systems shape the genetic diversity and population dynamics of arboviruses within insects, as demonstrated by mosquito-associated arboviruses, such as West Nile virus, chikungunya virus, and dengue virus (6–8).

In contrast to the dual-host transmission mode of arboviruses, ISVs replicate only in the insect host, without susceptibility to vertebrates or related cell lines (9–11). Taking mosquito-specific viruses (MSVs) as an example, due to the observed presence of these viruses in eggs, larvae, and male adult forms, it has been proposed that vertical transmission may play a major role in the maintenance of MSVs in nature (9, 11–13). However, direct experimental evidence of their vertical transmission is rare (14). In addition, horizontal transmission routes, such as the aquatic environmental contamination of larvae and plant nectar feeding, have been posited in some studies (15–17). Indeed, the specific mechanisms of MSV transmission and maintenance in nature are not yet clear, and all of these proposed transmission routes (or, more likely, mixed-mode transmission) are pending verification (12). Thus, an intriguing question is raised: do ISVs show tissue tropism within insects in a similar manner to arboviruses? The answer to this question will clearly be beneficial to deciphering the ISV maintenance mode in nature.

With the advancement of sequencing techniques in the last 2 decades, a large number of studies focusing on the insect virome have revealed the unprecedented diversity and abundance of ISVs (1, 18–20). Based on these findings, many ISVs have been discovered in viral families associated with arboviruses of medical significance, as indicated by phylogenetic analysis. It has been proposed that many arboviruses evolved from ISVs to infect vertebrates via adaptive evolution (12, 21). However, most of these studies were focused on the genetic diversity and evolution of these viruses. However, the tissue tropism and dynamics of ISVs within insect hosts remain largely unclear.

To this end, we performed whole-transcriptome sequencing targeting multiple tissues of two dominant mosquito species at three locations in Jiangsu Province in China. The results provided high-resolution virome profiles of different tissues, such as the midgut, salivary gland, legs, and eggs, in these mosquito species. In light of the current abundance of metasequencing data for mosquito species, the findings of this study provide new insight into the tissue tropism of mosquito-specific viromes.

## RESULTS

To elucidate the virome associated with natural conditions, we targeted the selected mosquito species in the field. In addition, wild-caught mosquitoes were transferred to the insectary for rearing to facilitate dissection and egg collection. Empirically, the wild-caught mosquitoes were difficult to rear for a long period in a man-made environment, and engorged mosquitoes showed greater longevity. In addition, the engorged mosquitoes were capable of laying eggs in most cases. Hence, we selected rural piggeries that did not apply insecticides as collection sites, where plentiful blood-engorged mosquitoes could be found. Finally, at three locations, we captured 454 engorged individual mosquitoes belonging to the two species and identified the viral communities present in pools of different tissues of these mosquitoes, including their eggs, midguts, salivary glands, legs, and the remainder of the carcass.

### Sample information.

Sample collection was conducted at the local piggeries of 3 sites (Yancheng, Liyang, and Yixing) within Jiangsu Province, China (see Fig. S1 in the supplemental material). The mosquito population of each piggery was dominated by a single species (Yancheng, Anopheles sinensis, *n* = 121; Liyang, *An. sinensis*, *n* = 169; Yixing, Culex quinquefasciatus, *n* = 164). Ultimately, individuals of two mosquito species, *An. sinensis* and *Cx. quinquefasciatus*, with engorged status were targeted for investigation. After being reared in a laboratory environment for 6 to 10 days, 60 individual mosquitoes from each location were picked and directly subjected to dissection without colony establishment. General information on the sampling and pools is provided in Table 1.

**TABLE 1:** Sample and library information of this study

Library^*a*^	Tissue	No. of:	
		Clean reads	Contigs^*b*^
*An. sinensis*			
Yancheng (*n* = 60)			
AM1	Midgut	42,103,238	154,711
AL1	Leg	53,363,544	245,382
AE1	Egg	56,427,152	772,305
AS1	Salivary gland	42,975,836	234,513
AC1	Remainder of carcass	39,423,904	119,181
Liyang (*n* = 60)			
AM2	Midgut	38,053,152	148,675
AL2	Leg	48,224,980	192,253
AE2	Egg	42,921,732	567,530
AS2	Salivary gland	54,918,096	244,611
AC2	Remainder of carcass	47,747,656	131,016
*Cx. quinquefasciatus*			
Yixing (*n* = 60)			
CM	Midgut	62,936,086	427,634
CL	Leg	58,130,744	615,936
CS	Egg	53,184,620	444,123
CE	Salivary gland	44,667,852	395,299
CC	Remainder of carcass	65,671,432	706,875

a The libraries are organized by mosquito number and location. In the library name, the first letter indicates the mosquito species (A, *An. sinensis*; C, *Cx. quinquefasciatus*), and the second letter indicates the tissue type (M, midgut; L, leg; E, egg; S, salivary gland; C, the remainder of the carcass).

b The numbers of contigs were based on the contigs assembled by the Trinity program.

### Library composition and virus abundance.

**FIG 1:**
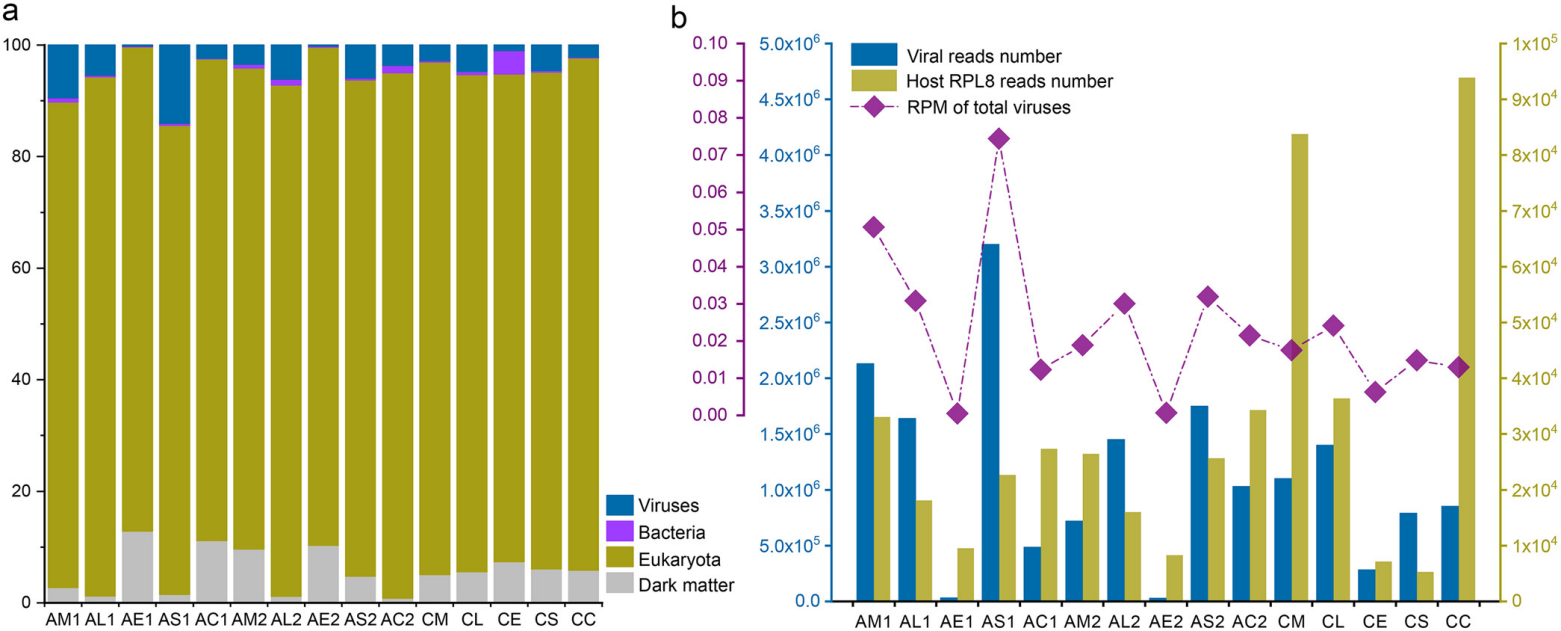
Overview of the sequencing data and composition. (a) Proportions of assembled contigs from different superkingdoms. “Dark matter” refers to the sequences that were unclassified, including a tiny proportion of sequences from *Archaea*. (b) Histogram displaying the read number of viruses and the host RPL8 gene in different pools. The dotted line indicates the reads per million reads (RPM) values of the viromes in different pools. The pools are named as follows. The first letter indicates the species (A, *An. sinensis*; C, *Cx. quinquefasciatus*), the second letter indicates the tissue type (M, midgut; L, leg; S, salivary gland; E, egg; C, the remainder of the carcass), and the number indicates the location (1, Yancheng; 2, Liyang). All of the *Cx. quinquefasciatus* mosquitoes were from Yixing.

A total of 15 libraries of transcriptomes with rRNA deleted representing different tissues of two mosquito species were characterized. RNA sequencing of these libraries resulted in 4.21 × 10^8^ to 6.56 × 10^8^ clean reads among these pools, which were *de novo* assembled into 1.55 × 10^5^ to 7.72 × 10^5^ contigs (Table 1). Subsequent BLASTx analysis by using Diamond revealed that the majority of the contigs (over 77%) belonged to Eukaryota, which were mainly associated with the transcripts of mosquito hosts. Reads associated with viruses in each pool accounted for 0.15% to 14.01% of the clean reads (mean, 4.38%). The egg pools of both mosquito species possessed the lowest proportion of viral reads among all tissue types (Fig. 1a). The read number of the host ribosomal protein L8 (RPL8) gene ranged from 8.3 × 10^3^ to 3.43 × 10^4^ in the tissue pools of *An. sinensis* and 5.3 × 10^3^ to 9.39 × 10^4^ in *Cx. quinquefasciatus*. For viruses, the read numbers ranged from 2.92 × 10^4^ to 3.20 × 10^6^ in *An. sinensis* and 2.81 × 10^5^ to 1.40 × 10^6^ in *Cx. quinquefasciatus*. Markedly more viral reads were found in the salivary glands of *An. sinensis* from both sites than in other tissues. In contrast, the eggs of *An. sinensis* showed the lowest viral richness (Fig. 1b).

### Virome comparisons among different types of tissues.

**FIG 2:**
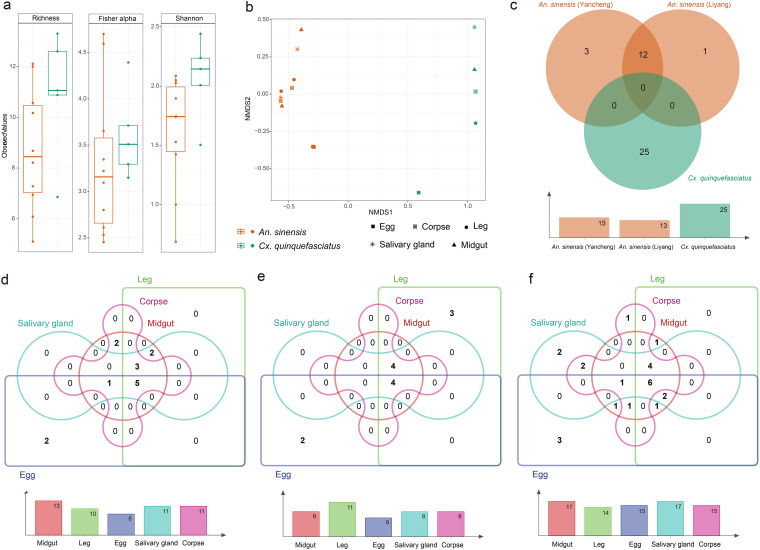
Comparison and connectivity of the viromes in the tissue pools of *An. sinensis* and *Cx. quinquefasciatus*. (a) Alpha diversity of viruses in the two mosquito species at the viral species level; (b) nonmetric multidimensional scaling (NMDS) of viruses at the viral species level; (c) Venn diagram presenting the relationships among the virome compositions of mosquitoes at three locations; (d to f): Venn diagrams presenting the relationships of the virome compositions among various tissue pools of *An. sinensis* from locations 1 and 2 and *Cx. quinquefasciatus*. The histograms below the Venn diagrams represent the number of viral species in various mosquito species or tissue pools.

There were no significant differences in the alpha diversity indices of *Cx. quinquefasciatus* and *An. sinensis*, possibly due to the limited sample size, although those of *Cx. quinquefasciatus* were greater (Fig. 2a). On the other hand, the nonmetric multidimensional scaling (NMDS) plot based on Bray-Curtis dissimilarities calculated using the normalized abundance of viral species revealed an obvious separation of viral communities based on the mosquito species (Fig. 2b), suggesting that the viromes harbored by the two mosquito species were distinctive. This was also reflected in the observation that no virus species were shared between the two mosquito species, whereas major overlap was observed between the samples of *An. sinensis* from different locations (Fig. 2c). In contrast to the tissue distribution observed in the host species, the viral communities were not separated based on tissue types (Fig. 2b). Indeed, some “core” viruses (*An. sinensis* in YanCheng, *n* = 5; *An. sinensis* in LiYang, *n* = 4; *Cx. quinquefasciatus*, *n* = 6) were ubiquitous in all tissues, and the intersection among the tissues, with the exception of eggs (3, 4), was also remarkable (Fig. 2d to f).

**FIG 3:**
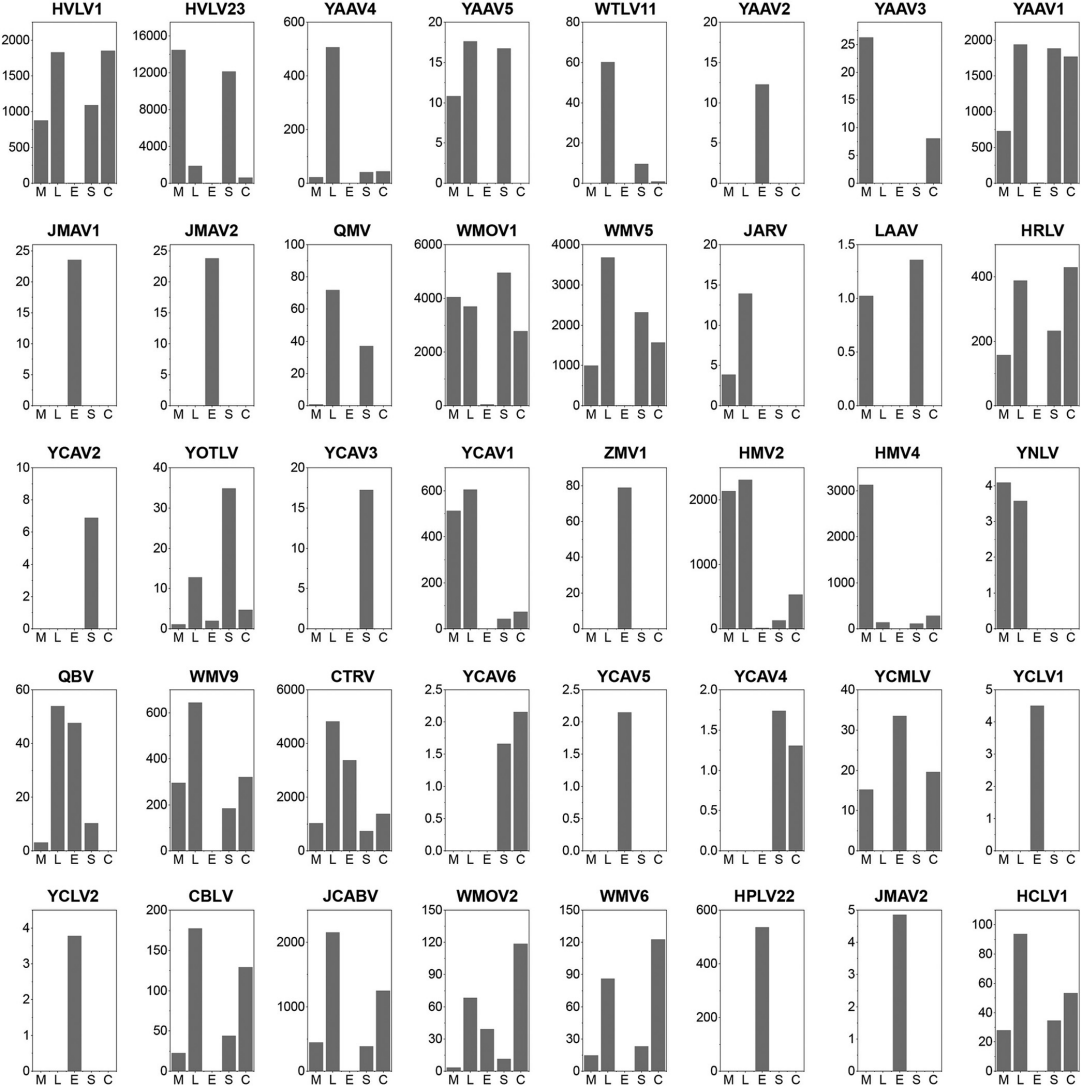
RPM values of each virus in different tissue pools. The data are displayed as the average values for *An. sinensis* from the two locations.

**FIG 4:**
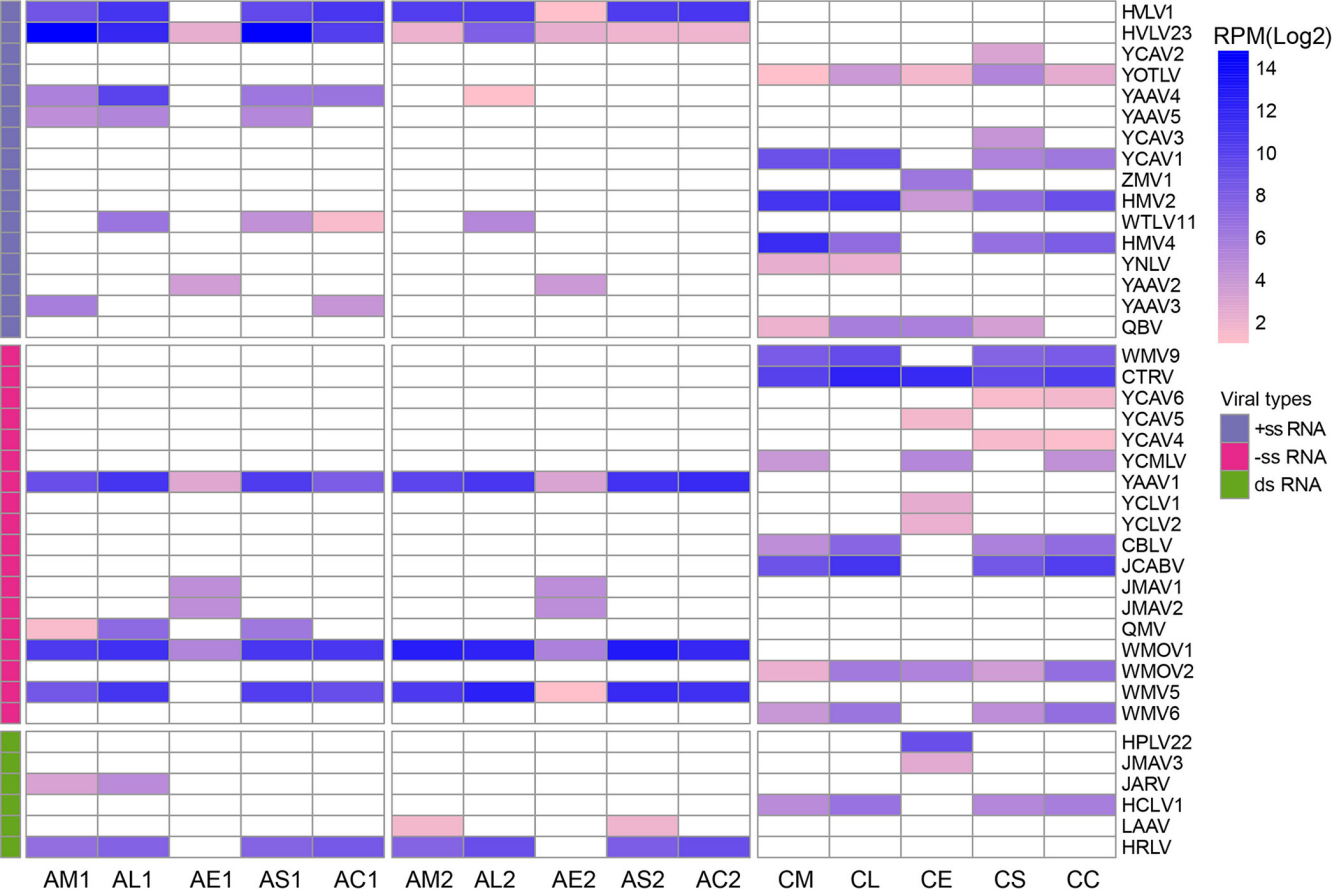
The heat map displays the RPM values of each virus in different tissue pools under the log_2_ function. In the pool names, the first letter indicates the species (A, *An. sinensis*; C, *Cx. quinquefasciatus*), the second letter indicates the tissue type (M, midgut; L, leg; S, salivary gland; E, egg; C, the remainder of the carcass), and the number indicates the location (1, Yancheng; 2, Liyang).

We then examined the dynamics of each individual viral species. A total of 40 viral species were identified in this study, 19 of which merited the assignment of new species using a species demarcation criterion of >10% amino acid divergence in the RNA-dependent RNA polymerase (RdRp) region (1). The detailed information and potential species names and abbreviations are listed in Table 2. The abundance of each detected virus was estimated and compared across different tissue pools (Fig. 3). The majority of the viruses identified here showed systemic infection (≥2 tissue types), and some (i.e., HVLV1, HVLV23, YOTLV, HMV2, CTRV, YAAV1, WMOV1, WMOV2, and WMV5) appeared in all tissue types. Interestingly, eggs represented a distinct type of tissue in our analyses: some viruses (e.g., YAAV1, YAAV2, YCLV1, YCLV2, JMAV1, JMAV2, HPLV22, and JMAV3) appeared only in eggs, whereas others (e.g., YAAV4, YCAV1, HMV4, WMV9, CBLV, JCABV, WMV6, HCLV1, and HRLV) were ubiquitous in all tissues except for eggs (Fig. 4).

**TABLE 2:** Information on the viruses detected in this study and their sequences

Viral species and strain	Abbreviation^*a*^	Reference virus (% protein identity)	Taxonomic group	Sequence(s) acquired^*b*^	GenBank accession no.
Hubei virga-like virus 1 strain LY882	HVLV1	Hubei virga-like virus 1 (98.78)	*Alsuviricetes*	Complete	MW452292
Hubei virga-like virus 23 strain YC066	HVLV23	Hubei virga-like virus 23 (99.81)	*Alsuviricetes*	Complete	MW452293
Yixing *Culex* associated virus 2 strain YX384	YCAV2	Probopyrinella latreuticola Nege-like virus (30.24)	*Alsuviricetes*	Partial	MW452295
Yixing originated *Tymoviridae*-like virus strain YX377	YOTLV	*Culex* originated *Tymoviridae*-like virus (93.69)	*Alsuviricetes*	Partial	MW452308
Yancheng *Anopheles* associated virus 4 strain YC505	YAAV4	Brandeis virus (36.45)	*Alsuviricetes*	Complete	MW452316
Yancheng *Anopheles* associated virus 5 strain YC512	YAAV5	Muthill virus (36.94)	*Alsuviricetes*	Complete	MW452317
Yixing *Culex* associated virus 3 strain YX384	YCAV3	Tropical soda apple mosaic virus (30.08)	*Alsuviricetes*	Partial	MW452296
Yixing *Culex* associated virus 1 strain YX683	YCAV1	Hubei virga-like virus 23 (43.90)	*Alsuviricetes*	Complete	MW452294
Zhejiang mosquito virus 1 strain YC616	ZMV1	Zhejiang mosquito virus 1 (99.49)	*Picornavirales*	Complete	MW452304
Hubei mosquito virus 2 strain YX512	HMV2	Hubei mosquito virus 2 (100)	*Tombusviridae*	Partial	MW452307
Wenzhou tombus-like virus 11 strain YC895	WTLV11	Wenzhou tombus-like virus 11 (99.42)	*Tombusviridae*	Complete	MW452314
Hubei mosquito virus 4 strain YX487	HMV4	Hubei mosquito virus 4 (99.42)	*Tombusviridae*	Complete	MW452315
Yixing narna-like virus strain YX788	YNLV	Hubei narna-like virus 17 (70.37)	*Narnaviridae*	Complete	MW452310
Yancheng *Anopheles* associated virus 2 strain YC745	YAAV2	Wilkie narna-like virus 2 (34.99)	*Narnaviridae*	Complete	MW452311
Yancheng *Anopheles* associated virus 3 strain YC093	YAAV3	Wilkie narna-like virus 2 (45.81)	*Narnaviridae*	Complete	MW452312
Quang Binh virus strain YX594	QBV	Quang Binh virus (99.73)	*Flavivirus*	Complete	MW452275
Wuhan mosquito virus 9 strain YX073	WMV9	Wuhan mosquito virus 9 (99.01)	*Monjiviricetes*	Complete	MW452302
Culex tritaeniorhynchus rhabdovirus strain YX204	CTRV	Culex tritaeniorhynchus rhabdovirus (99.77)	*Monjiviricetes*	Complete	MW452298
Yixing *Culex* associated virus 6 strain YX363	YCAV6	Wenling dimarhabdovirus 8 (37.11)	*Monjiviricetes*	Partial	MW452301
Yixing *Culex* associated virus 5 strain YX195	YCAV5	Formica exsecta virus 4 (51.36)	*Monjiviricetes*	Partial	MW452300
Yixing *Culex* associated virus 4 strain YX281	YCAV4	Drosophila melanogaster sigmavirus (29.33)	*Monjiviricetes*	Partial	MW452299
Yixing Culex monjiviricetesnega-like virus strain YX518	YCMLV	Culex monjiviricetesnega-like virus 2 (71.33)	*Monjiviricetes*	Complete	MW452297
Yancheng *Anopheles* associated virus 1 strain YC899	YAAV1	Xincheng mosquito virus (96.69)	*Monjiviricetes*	Complete	MW452303
Yixing chuvirus-like virus 1 strain YX740	YCLV1	Imjin mivirus (46.78)	*Monjiviricetes*	Partial	MW452289
Yixing chuvirus-like virus 2 strain YX794	YCLV2	Wenling crustacean virus 13 (29.06)	*Monjiviricetes*	Partial	MW452290
*Culex Bunyavirales*-like virus strain YX771	CBLV	*Culex Bunyavirales*-like virus (99.45%)	*Bunyavirales*	Complete	MW452278
Jiangsu *Culex* associated *Bunyaviralesvirus* strain YX392	JCABV	Culex pseudovishnui *Bunyavirales*-like virus (65.92)	*Bunyavirales*	Complete	MW452279
Jiangsu mosquito associated virus 1 strain YC673	JMAV1	Jiangxia mosquito virus 1 (29.96)	*Bunyavirales*	Partial	MW452280
Jiangsu mosquito associated virus 2 strain YC990	JMAV2	Jiangxia mosquito virus 1 (69.66)	*Bunyavirales*	Partial	MW452281
Qingnian mosquito virus strain YC179	QMV	Qingnian mosquito virus (99.38)	*Bunyavirales*	Complete	MW452282
Wuhan mosquito orthophasmavirus 1 YC603	WMOV1	Wuhan mosquito orthophasmavirus 1 (99.84)	*Bunyavirales*	Complete	MW452283
Wuhan mosquito orthophasmavirus 2 YX337	WMOV2	Wuhan mosquito orthophasmavirus 2 (98.02)	*Bunyavirales*	Complete	MW452284
Wuhan mosquito virus 5 strain LY278	WMV5	Wuhan mosquito virus 5 (99.62)	*Orthomyxoviridae*	Complete	MW452276
Wuhan mosquito virus 6 strain YX592	WMV6	Wuhan mosquito virus 6 (99.87)	*Orthomyxoviridae*	Complete	MW452277
Hubei partiti-like virus 22 strain YX195	HPLV22	Hubei partiti-like virus 22 (100)	*Durnavirales*	Partial	MW452285
Jiangsu mosquito associated virus 3 strain YX356	JMAV3	Wuhan spider virus 10 (94.75)	*Durnavirales*	Complete	MW452286
Jiangsu *Anopheles* related virus strain YC382	JARV	Diatom colony-associated dsRNA virus 11 (32.92)	*Ghabrivirales*	Complete	MW452287
Hubei chryso-like virus 1 strain YX526	HCLV1	Hubei chryso-like virus 1 (99.19)	*Ghabrivirales*	Partial	MW452288
Liyang *Anopheles* associated virus 1 LY791	LAAV	High Island virus (54.27)	*Reoviridae*	Partial	MW452305
Hubei reo-like virus strain LY159	HRLV	Hubei reo-like virus 12 (99.86)	*Reoviridae*	Complete	MW452306

a In order to provide a concise description, the virus names in this study were presented as abbreviations throughout the text.

b The sequences here refer to the RNA replicase gene that was applied for the phylogenetic analysis in this study.

**FIG 5:**
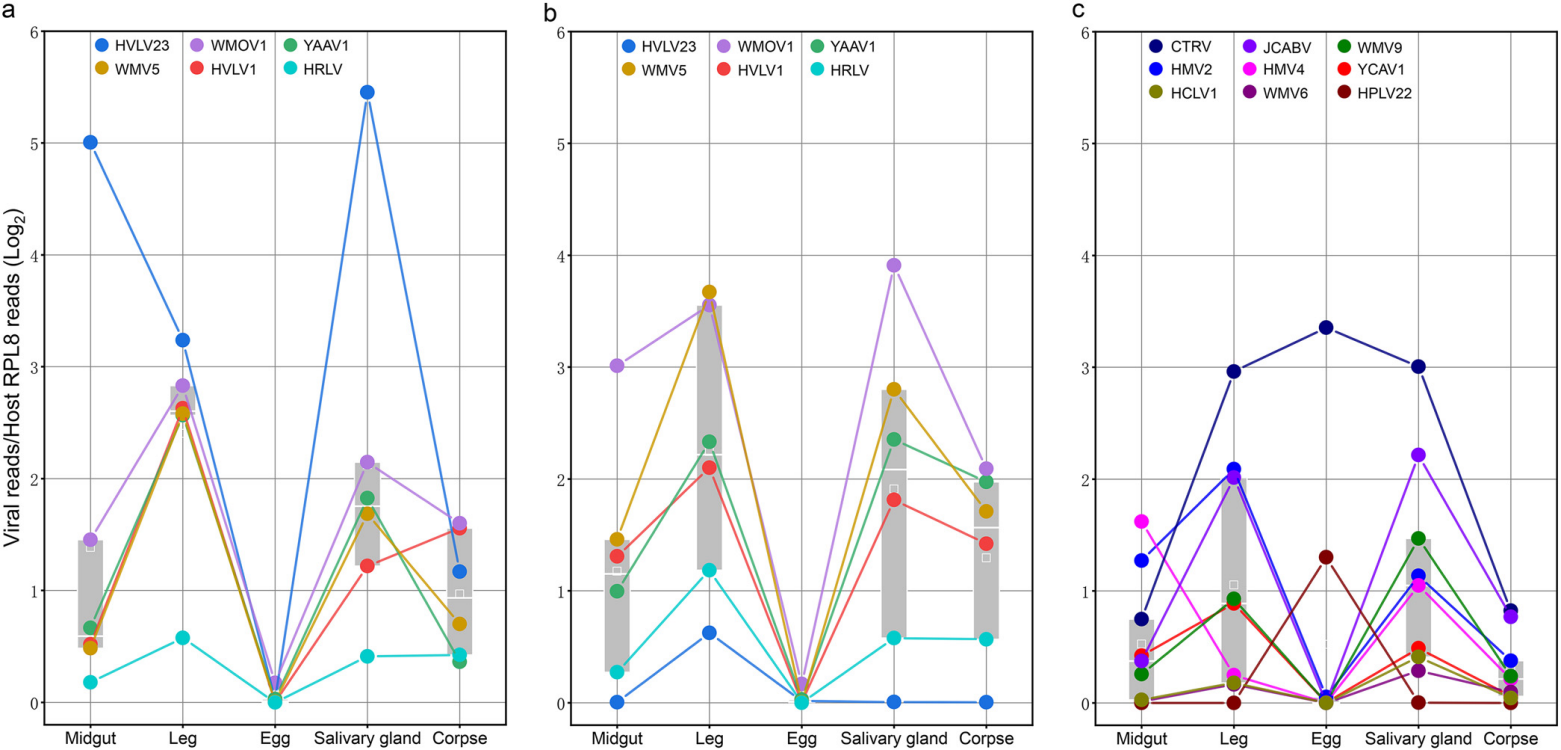
Box plot of the viral read number dynamics of dominant viral species among different mosquito tissue pools. Each colored dot indicates the viral reads normalized using the host RPL8 gene under the log_2_ function in different tissue pools. To reveal the dynamic trend with a visualized range, the viral read/RPSL8 ratio was adjusted by adding 1. Only the dominant viral species were considered. The box indicates the 25 to 75% range of normalized viral reads of each pool. The white line within the box indicates the median, and the white square indicates the mean value. Panels a, b, and c present the results for mosquitoes from Yancheng, Liyang, and Yixing, respectively.

To compare the abundance of some dominant viruses in different tissues, 15 viruses (*An. sinensis*, *n* = 6; *Cx. quinquefasciatus*, *n* = 9) were selected, and their read numbers were normalized against the read counts of the corresponding host RPL8 gene (Fig. 5). On average, the legs and salivary glands showed the highest abundance, followed by the midgut and carcass, while eggs showed the lowest abundance in both *An. sinensis* and *Cx. quinquefasciatus*.

### Phylogenetic and tissue tropism features of the identified viruses.

**(i) Positive-stranded RNA viruses.** A total of 16 positive-stranded RNA viruses were identified. These viruses belonged to 5 major taxonomic virus groups, as indicated by other studies (1, 22): *Alsuviricetes* (HVLV1, HVLV23, YCAV2, YOTLV, YAAV4, YAAV5, YCAV3, and YCAV1), *Picornavirales* (ZMV1), *Solemoviridae*-*Tombusviridae* (HMV2, WTLV11, and HMV4), *Narnaviridae* (YNLV, YAAV2, and YAAV3), and *Flavivirus* (QBV).

**FIG 6:**
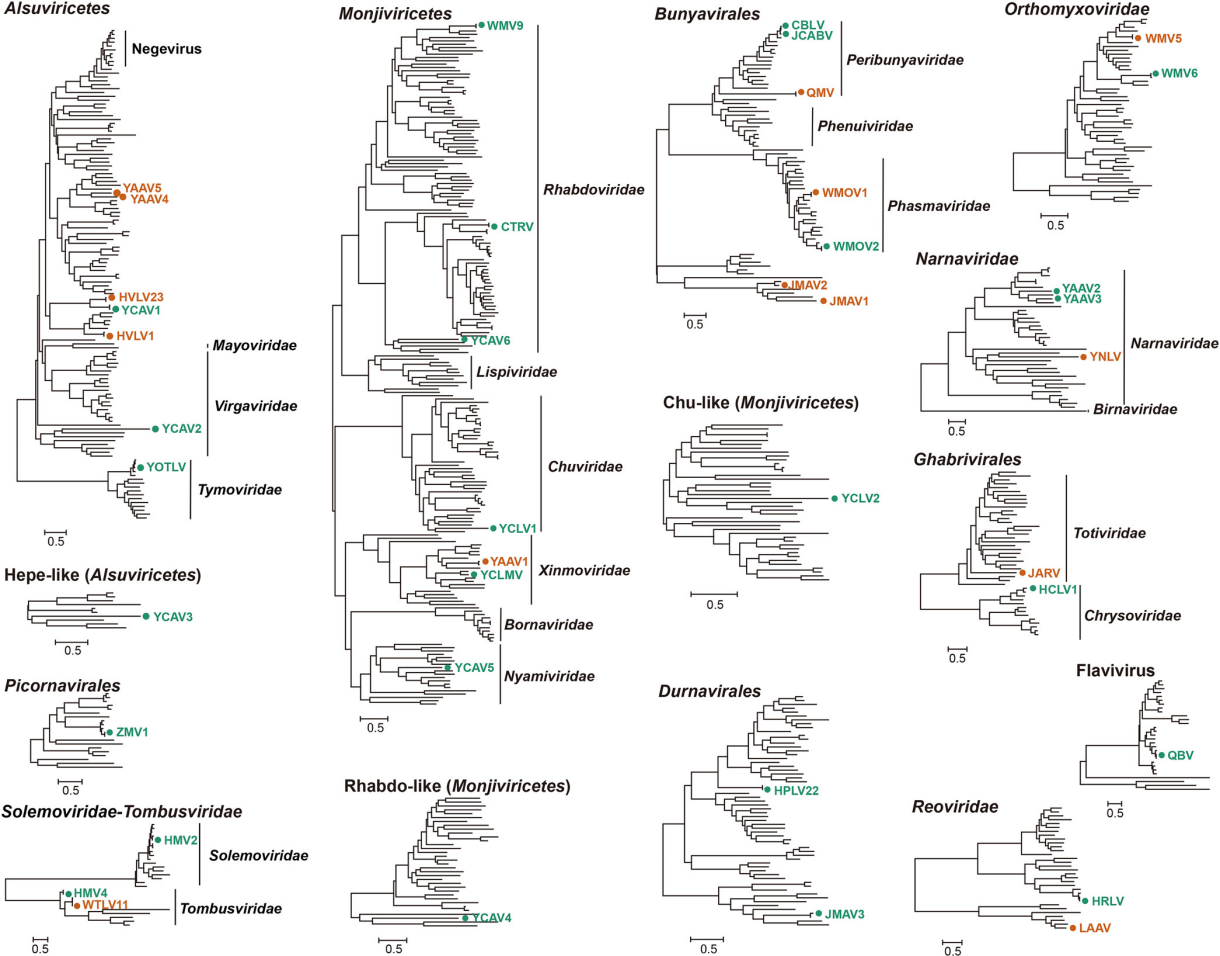
Phylogeny of the detected RNA viruses with related references in different taxonomic groups. The annotation is as follows. In the dots and viral names, orange indicates the viruses detected in *An. sinensis*, and green indicates those in *Cx. quinquefasciatus*. Due to the incongruous alignment resulting from some partial sequences, some phylogenetic trees of the same taxonomic status were constructed separately for the coordinated topology (such as Hepe-like, Chu-like, and Rhabdo-like).

Among the viruses belonging to the *Alsuviricetes*, YOTLV and YCAV2 were clustered within *Tymoviridae* and *Virgaviridae*, respectively, while others were unclassified based on the current viral taxonomy. The HVLV1 and HVLV23 viruses showed high abundance in all *An. sinensis* tissue pools and clustered with other viruses from different insect hosts, including YCAV1. YAAV4 and YAAV5 were two novel viruses that formed a new linage and fell into a large clade of viruses with a wide range of hosts, such as *Scaptodrosophila* and *Entomophthora* species. Unlike the viruses that showed multiple-tissue tropism, two novel viruses, YCAV2 and YCAV3, were detected only in the salivary gland pools, and they fell within the clade associated with plant hosts. ZMV1 was found exclusively in the egg pools of *Cx. quinquefasciatus*. It was the only virus of the order *Picornavirales* that clustered with other unclassified viruses. HMV2, detected in all tissue pools of *Cx. quinquefasciatus*, was previously identified in mosquitoes of Hubei Province, China. This virus was similar to a group of viral strains potentially belonging to *Solemoviridae* identified in various mosquito species. The two viruses putatively belonging to family *Tombusviridae*, WTLV11 and HMV4, were detected in *An. sinensis* and *Cx. quinquefasciatus*, respectively. Notably, the tissue tropism of WTLV11 differed between the *An. sinensis* individuals from the two sampling locations, and this virus has reportedly also been identified in some *Culex* species, such as *Cx. tritaeniorhynchus* (23). Among the viruses from the narna-like group, YNLV, YAAV2, and YAAV3 were all novel and fell within different clades of *Narnaviridae* in the phylogenetic tree. Whether these viruses were detected in *An. sinensis* or *Cx. quinquefasciatus*, all of the viruses were detected in limited tissue types at low abundance. QBV, a previously reported mosquito-specific virus, was detected in all tissue pools of *Cx. quinquefasciatus* and clustered with other Culex-specific viruses within the genus *Flavivirus* (24) (Fig. 6; Fig. S2a).

### (ii) Negative-stranded RNA viruses.

A total of 18 negative-stranded RNA viruses belonging to 3 taxonomic virus groups were identified. These groups were *Monjiviricetes* (WMV9, CTRV, YCAV6, YCAV5, YCAV4, YCMLV, YAAV1, YCLV1, and YCLV2), *Bunyavirales* (CBLV, JCABV, JMAV1, JMAV2, QMV, WMOV1, and WMOV2), and *Orthomyxoviridae* (WMV5 and WMV6).

The viruses from *Mononegavirales* were divergent from each other and fell within four viral family clades in the phylogenetic tree, including *Rhabdoviridae*, *Chuviridae*, *Xinmoviridae*, and *Nyamiviridae*. Four viruses, WMV9, CTRV, YCAV6, and YCAV4, exhibited genetic features of rhabdovirus or rhabdovirus-like RdRp, representing a viral group associated with highly divergent hosts. Notably, CTRV was detected in all tissue pools with high abundance, especially in eggs, but WMV9 was not found in the egg pool, even though it showed high abundance in other tissues. YCAV5 was novel and fell within a distinct family clade, *Nyamiviridae*. Two viruses, YCMLV and YAAV1, were closely related to anpheviruses within the family *Xinmoviridae*. Two viruses, YCLV1 and YCLV2, were found only in the egg pools; they were phylogenetically included within *Jingchuvirales*, a viral order that composed of chuviruses and some other unclassified viruses. Apart from YAAV1, all the viruses assigned to *Mononegavirales* in this study were identified in the tissue pools of *Cx. quinquefasciatus*. Among the viruses belonging to the order *Bunyavirales*, CBLV, JCABV, and QMV fell within the family *Peribunyaviridae*, and WMOV1 and WMOV2 fell within the family *Phasmaviridae*. With the exception of JCABV, which was newly identified in this study, the other four viruses were closely related to viruses reported elsewhere (1, 25). CBLV and JCABV were detected in all tissues of *Cx. quinquefasciatus* except the eggs. WMOV1 and WMOV2 were found in all tissues of *An. sinensis* and *Cx. quinquefasciatus*, respectively. Two novel viruses, JMAV1 and JMAV2, which were detected only in the egg pools of *An. sinensis*, clustered with some other unclassified viruses within a unique clade in the phylogeny of the order *Bunyavirales*. Two viruses, WMV5 and WMV6, were identical to two unclassified viruses in *Orthomyxoviridae* (1). WMV5 was detected in all the tissue pools of *An. sinensis*, and WMV6 was detected in all the tissue pools except the eggs of *Cx. quinquefasciatus* (Fig. 6; Fig. S2b).

### (iii) Double-stranded RNA viruses.

We identified six double-stranded RNA viruses belonging to three viral groups: *Durnavirales* (HPLV22 and JMAV3), *Ghabrivirales* (JARV and HCLV1), and *Reoviridae* (LAAV and HRLV). HPLV22 and JMAV3 were both detected exclusively in the egg pools of *Cx. quinquefasciatus*. HPLV was identical to a reported strain previously detected in mosquitoes, while JMAV3 was closely related to a strain from spiders (1). JARV was novel and phylogenetically clustered with strains belonging to *Totiviridae*, a family of viruses hosted by some protozoa and fungi. Conversely, HCLV1 was closely related to some mosquito viruses within family *Chrysoviridae*. These two ghabriviruses were detected in some different tissue types of *Anopheles* and *Culex*. HRLV was identical to a mosquito virus and clustered with some other viruses from different insects. LAAV was novel and clustered with some other ISVs. Interestingly, HRLV and LAAV were both detected in the tissue pools except for the eggs of *Cx. quinquefasciatus* and *An. sinensis*, respectively (Fig. 6; Fig. S2c).

## DISCUSSION

In comparison to *An. sinensis*, *Cx. quinquefasciatus* has a virome with greater variety but lower abundance. In the viral taxonomy of individuals, the virus community identified in *Cx. quinquefasciatus* was dominated by viruses within the orders *Mononegavirales* and *Bunyavirales*, while the viruses found in *An. sinensis* showed a scattered distribution among various viral groups. Considering the sampling sites, 75% of the viral species were shared between *An. sinensis* from both locations. This was consistent with the results of some other studies showing that a given mosquito species possesses a stable virome regardless of sampling sites (22, 26). Phylogenetically, none of the viruses discovered in this study were closely related to existing arboviruses, but the majority clustered with various ISVs, whether or not they were hosted by mosquitoes. The viruses showing high divergence from existing viruses all fell within the viral group containing ISVs.

Despite a dearth of relevant evidence, we infer that ISVs found within mosquitoes, like arboviruses, show tissue-specific replication preferences. The viruses revealed in this study presented tissue tropism within the mosquitoes, as expected, and unique features were observed among different viruses. Viruses that showed systematic infection in *An. sinensis* were typically dominant, and a general dynamic trend was found among different tissues: eggs showed the lowest viral abundance, while legs and salivary glands showed the highest viral richness. The same tendency was observed among the dominant viruses of *Cx. quinquefasciatus*, although there were some exceptions (CTRV, WMV6, and HMV4). Research on Aedes albopictus derived from both laboratory and field conditions at various life stages (eggs, larvae, pupae, and adults) has revealed a “vertically transmitted core virome” set that is stable throughout all stages (26). Analogously, in terms of tissue tropism, the dominant viruses observed in this research can also be regarded as a “core virome.” In general, the tissue-tropism-based core virome refers to those viruses that are prevalent in all tissues, but with various levels of abundance. The present study also demonstrated that the core viruses found in *An. sinensis* from both locations, such as YAAV1, WMOV1, WMV5, and HRLV, were detected at the highest abundance in the legs and salivary glands, followed by the midgut and remainder of the carcass, and showed the lowest abundance in the eggs. On the other hand, those viruses that were not included in the “core virome,” such as YAAV5, QMV, JARV, and LAAV, were recovered only in one location and with low abundance.

The salivary gland is considered the tissue site where the genetic diversity of arboviruses is replenished under the selection pressure of a dual-host shift (11, 27–30). Strikingly, as demonstrated in this study, the salivary gland is a tissue type harboring a viral community with high diversity. Relative to the eggs, the abundance of the virome in the salivary glands of the two mosquito species was considerable. Thus, the salivary gland of mosquitoes potentially serves as a significant amplification site for a wide range of viruses, whether arboviruses or ISVs. This study lends credence to the long-standing hypothesis that ISVs might be exploited as biological control agents to prevent pathogenic arbovirus infection (12, 31, 32). For this reason, the viruses identified in this study that replicate preferentially in the salivary glands, such as HVLV23 and CTRV, may play a role as candidate arbovirus competitors. The virome harbored by the eggs of the two mosquito species is distinct from that in other tissues in either composition or abundance. It has been reported that some Aedes albopictus-associated viruses with vertical transmission start with a low concentration at the earliest developmental stage (larvae), followed by amplification in subsequent stages (pupae and adult) (26). This was indirectly proven by the discrepancy in the abundance of most core viruses between eggs and other tissues presented in this study. Additionally, the present study reveals viruses detected exclusively in eggs for the first time, indicating the existence of a unique viral group without amplification in subsequent developmental stages—at least in female adults. In studies of arboviruses, the viruses identified in mosquito legs are generally regarded as markers of disseminated infection within the mosquito body (33, 34). However, the present study showed that viral abundance in the legs was higher than that in other tissues. Thus, there are two possibilities that may explain the specific identification of ISVs in the leg: (i) active viral replication and amplification occur in the legs, and/or (ii) viruses are disseminated to the legs. Given that the viral load in the legs is relatively high, both phenomena probably occur simultaneously. Regardless, whether the leg is an amplification site or leg detection is just a marker of disseminated infection needs to be validated experimentally.

Vertical transmission is believed to be the primary route of ISV transmission among mosquito populations, although this is based on limited experimental evidence, and other mechanisms are assumed to supplement it (10, 13). Due to the prevalence of the core virome in various tissues, especially in eggs, it is reasonable to deduce that these viruses show vertical transmission. Thus, the likely maintenance mode of the core virome may be deciphered. As ISVs, without vertebrate hosts in their transmission cycle, these viruses potentially enter the mosquito's body along with sugar sources, such as nectar and honeydew, rather than via blood meals (35). Then, the diverticula may be the initial amplifying sites of these viruses. Subsequent infection dissemination may be reinforced via amplification in other tissues, such as the salivary gland, legs, and midgut, as previously noted. Despite the low abundance in eggs, the dissemination of the infection to eggs allows vertical transmission. In this process, the involvement of other factors, such as the contamination of aquatic habitats or plants or male mosquitoes, is worthy of attention. Additionally, the distinctiveness of the tissue tropism of some viruses may reflect the unique feature of their maintenance mode. CTRV, identified in *Cx. quinquefasciatus*, represents a unique case since it was detected in high abundance in the eggs as well as in the legs and salivary glands. Thus, CTRV is definitely characterized by a maintenance mode involving vertical transmission, while showing disparate tissue tropism compared with other viruses. HVLV23 was notable because of its contrasting tissue tropism between *An. sinensis* from the two sampling locations. With the exception of the egg pools, the abundance of this virus in other tissue pools, especially in the salivary gland, midgut, and legs of *An. sinensis*, was significantly higher in Yancheng than in Liyang. Therefore, beyond vertical transmission, the transmission of HVLV23 is probably determined by other factors, such as the aquatic habitat or routes associated with male mosquitoes. Analogously, the viruses identified in *An. sinensis*, notably YAAV4, YAAV5, YCTLV, and YAAV3, presented a variety of distinct maintenance modes associated with environmental factors. For the viruses that were found only in the eggs, it is likely that male mosquitoes play a role in their transmission through the mating process.

Although additional data are needed to confirm the relationship between tissue tropism and phylogenetic traits, certain relevant intriguing findings highlighted in this work are worth noting. In many situations, viruses with the same tissue tropism belong to the same taxonomic group. Three sets of viruses found exclusively in eggs were categorized as members of *Chuviridae* (YCLV1 and YCLV2), *Durnavirales* (HPLV22 and JMAV3), and unclassified bunyaviruses (JMAV1 and JMAV2). Although the viruses detected in this study from *Peribunyaviridae* and *Phasmaviridae* clustered within the bunyavirus group, peribunyaviruses (WMOV1 and WMOV2) were ubiquitous in all tissues, whereas the phasmaviruses (CBLV, JCABV, and QMV) were ubiquitous in all tissues except eggs. Due to the phylogenetic relationships and host tropism of ISVs and arboviruses, it is assumed that mammalian arboviruses evolved from ISVs into dual-host viruses, implying that new arboviruses may originate from ISVs (36, 37). The diverse tissue tropism of different viruses could indicate an interpretation of the evolutionary mechanism of mosquito-associated viruses from new perspectives, such as viral susceptibility and replication efficiency in various tissue cells. The core viruses suggested by the present study probably develop transmissibility to more types of tissue cells than those that replicate only in certain tissue types. Regardless, the acquisition of transmissibility to salivary glands is indispensable in the potential evolutionary pathway of arboviruses.

By serving as transmitters of several pathogens, mosquitoes greatly influence human health (35). Both *An. sinensis* and *Cx. quinquefasciatus* are significant disease vectors that are distributed across China (38). Regardless, it is important to note that the lack of technical replicates was an obvious limitation of this study due to the sampling constraint of collecting the mosquito species in their blood-feeding state. A broader sampling of mosquito fauna, such as *Aedes* species, would provide more dimensional data for this kind of research. In addition, if male mosquitoes are taken into consideration in future work, it may reveal an integrated maintenance mode of mosquito-specific viruses in their natural habitat. Furthermore, experimental validation is essential in the future to better confirm the transmission routes of individual viruses, especially those detected in the eggs and salivary glands.

Overall, our study used transcriptome sequencing to establish a high-resolution profile of the viromes hosted by two mosquito species, *Cx. quinquefasciatus* and *An. sinensis*. Unlike previous studies on mosquito viruses, we first examined the virus community, concentrating on different tissues within mosquitoes. Depending on the common features shared by some dominant viruses, such as their prevalence in all tissues with the same dynamics among tissues, we defined the core virome in terms of tissue tropism. Strikingly, this work showed that the salivary gland served as an important amplification site for the core viruses, while the eggs exhibited a distinct viral community with low abundance. The differences in tissue tropism provide clues about various transmission modes, providing a novel perspective for further research on the maintenance mode and evolutionary history of the virome harbored by mosquitoes.

## MATERIALS AND METHODS

### Field-engorged mosquito collection and dissection.

The dominant mosquito species at the selected sites were collected from 3 geographical locations (Yancheng, Liyang, and Yixing) in Jiangsu Province from June to August 2018. The blood-engorged mosquitoes were captured alive in rural piggeries and kept in mosquito cages. Briefly, collection was performed after 20:00, when the mosquitoes were most abundant in the piggeries. The blood-feeding status of their abdomens was confirmed prior to capture. Then, the mosquitoes were captured using a device that can catch an individual mosquito alive. During sampling, the captured mosquitoes were preliminarily pooled in different cages based on species morphology. Later, in the laboratory, the species was confirmed using the molecular identification approach described previously (39). Briefly, DNA was extracted from the whole mosquito, and the internal transcribed spacer 2 (ITS2) gene was amplified by using PCR with the reported primers. The amplicons were interpreted by using Sanger sequencing, on the basis of which further identification analysis was conducted. The mosquitoes were immediately transferred to the insectary and reared at the appropriate room temperature (26°C to 28°C) and relative humidity (75% ± 5%). Water and glucose were provided in the cage to ensure the essential nutrition for the sustenance of the field-captured mosquitoes. Additionally, a bowl with sterile water covered by a piece of filter paper was placed in the cage to collect the eggs laid by the mosquitoes.

These mosquitoes were reared for 6 to 10 days (*An. sinensis*: 6–7 days; *Cx*. *quinquefasciatus*: 9-10 days) to ensure that the blood meal in their abdomens had been fully digested and that they had enough time to lay eggs. During this period, the laid egg rafts were collected in a sterile tube with RNAlater solution (Invitrogen) and stored at −80°C. Thereafter, some of the reared mosquitoes were picked (60 individuals from each location) and anesthetized with ether before being dissected under the microscope. To reduce the impact of cross-contamination as much as possible, dissection was carried out by skilled staff using specific tools that were sterilized (with heat and alcohol) each time a tissue was picked. The eggs, midguts, salivary glands, legs, and remainder of the carcass of different individuals were pooled, such that each pool represented one tissue type of one mosquito species from one location.

### Library preparation and sequencing.

The pooled tissues of each mosquito group in RNAlater solution were concentrated via centrifugation at 12,000 × *g* for 5 min at 4°C, and the supernatant liquid was removed. Thereafter, the tissues were frozen using liquid nitrogen and homogenized with a mixer mill MM 400 (Retsch). Total RNA was then extracted using the RNeasy minikit (Qiagen) following the manufacturer’s instructions. Before library preparation, host rRNA was removed using a Ribo-Zero-Gold (human-mouse-rat) kit (Illumina). The sequencing libraries were constructed using the TruSeq total RNA library preparation protocol (Illumina). Paired-end (100-bp) sequencing was performed on the HiSeq 4000 platform (Illumina) and was carried out by BGI Tech.

### Virus discovery and bioinformatic analysis.

The obtained sequencing reads were first subjected to trimming and quality control by using Trimmomatic according to quality scores of >30 (40). The resulting clean reads were then *de novo* assembled using the Trinity program with the default parameter settings (41). The assembled contigs were first compared against the viral proteins downloaded from NCBI (NCBI txid:10239) using the Diamond BLASTx program in sensitive mode for taxonomic annotation (42). The E value threshold for diamond BLASTx analyses was set to 10E−5 to avoid false positives. Potential viral contigs were then compared against the entire nonredundant (nr) protein database to remove nonviral sequences. In particular, to eliminate potential endogenous viral elements (EVEs) in our viral sequences, we compared them to the Whole Genome Shotgun (WGS) database, limiting taxonomy to the family *Culicidae*.

The potential open reading frames (ORFs) of the newly obtained viruses were identified using ORF Finder. The functional domains associated with each ORF were identified by structural BLAST searches against the Conserved Domain Database (CDD). For the viruses with a segmented genome, various segments were identified based on homology and confirmed based on conserved genome termini, sequencing depth, coappearance with the RdRp segments, and phylogenetic analysis (1).

To determine the abundance of the RNA viral transcripts, the number of reads mapped to viral genomes or genes was estimated by using bowtie2 software (43). Specifically, the reads per million reads (RPM) values of the RNA polymerase gene were used to quantify viral abundance in various viruses. To avoid potential index hopping in the sequencing results, which may affect the estimation of abundance, we used a threshold of greater than 0.1% for the total reads associated with a single virus to be considered positive. In addition, the ribosomal protein L8 (RPL8) gene counts in the assembled contigs of *An. sinensis* or *Cx. quinquefasciatus* were also determined to indicate the abundance of the host gene. For the comparison of viruses in different tissue pools, the number of host RPL8 reads was used to normalize the read numbers of the detected viruses. The heat map showing the abundance of each virus species was generated using the pheatmap package implemented in R (44). The comparisons of virome diversity and alpha and beta diversity were calculated using the phyloseq package in R (45). The overlap of the virome species from different mosquito species (locations) in the tissues was demonstrated in Venn diagrams using the online tool Evenn (www.ehbio.com/test/venn/#/).

### Phylogenetic analysis.

The amino acid sequences of RdRp or PB1 proteins were used to determine the phylogenetic relationships of the newly detected viral species and strains with other reference species and strains. Based on the current ICTV report (https://talk.ictvonline.org/ictv-reports/ictv_online_report/), we established a phylogenetic tree of different virus orders. The alignment of the amino acid sequences was performed using the E-INS-i algorithm implemented in the MAFFT (version 7) program (46). Ambiguously aligned regions were removed by using TrimAl (47). The ProtTest 3.4 program was used to determine the best-fit model of amino acid substitutions (48). Maximum likelihood (ML) phylogenetic trees were reconstructed using PhyML, utilizing the best-fit substitution model. Individual node support was evaluated using an approximate likelihood ratio test (aLRT) and an SH-like procedure implemented in PhyML (49). The trees were subsequently edited and visualized by the tree editor of MEGA7 software (50).

### Data availability.

The sequence reads of each tissue pool in this study are available in the NCBI Sequence Read Archive (SRA) database under BioProject accession no. PRJNA716469.
