# Supplementary material for: Deciphering the Tissue Tropism of the RNA Viromes Harbored by Field-Collected Anopheles sinensis and Culex quinquefasciatus
Source: Microbiol Spectr. 2022 Aug 15;10(5):e01344-22. doi: 10.1128/spectrum.01344-22 (PMC9604083; doi:10.1128/spectrum.01344-22)
Supplement: Supplemental file 1 — Fig. S1 and S2. Download spectrum.01344-22-s0001.pdf, PDF file, 4.7 MB [file spectrum.01344-22-s0001.pdf]

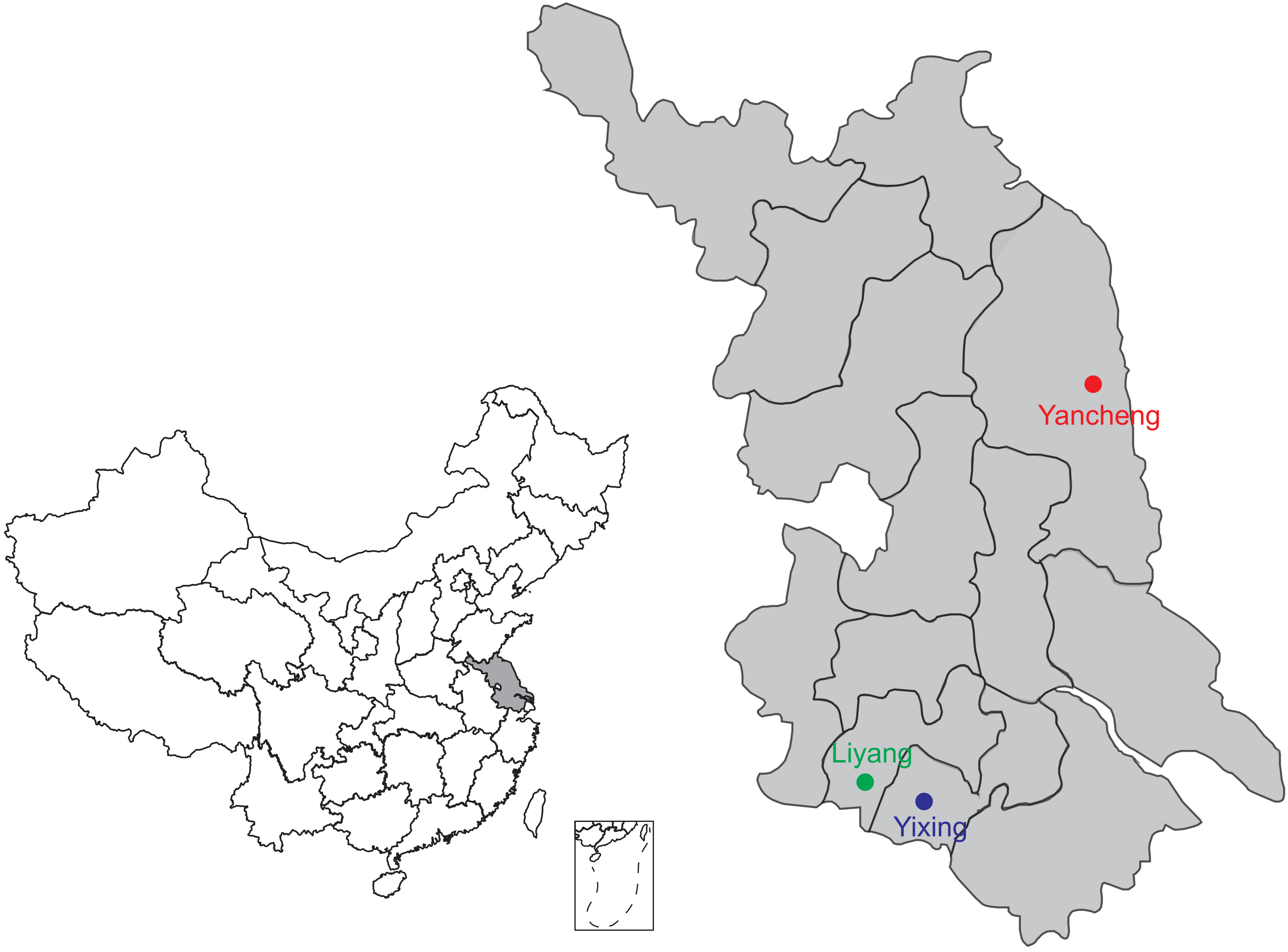

Figure S1. Map of the sampling sites in this study.

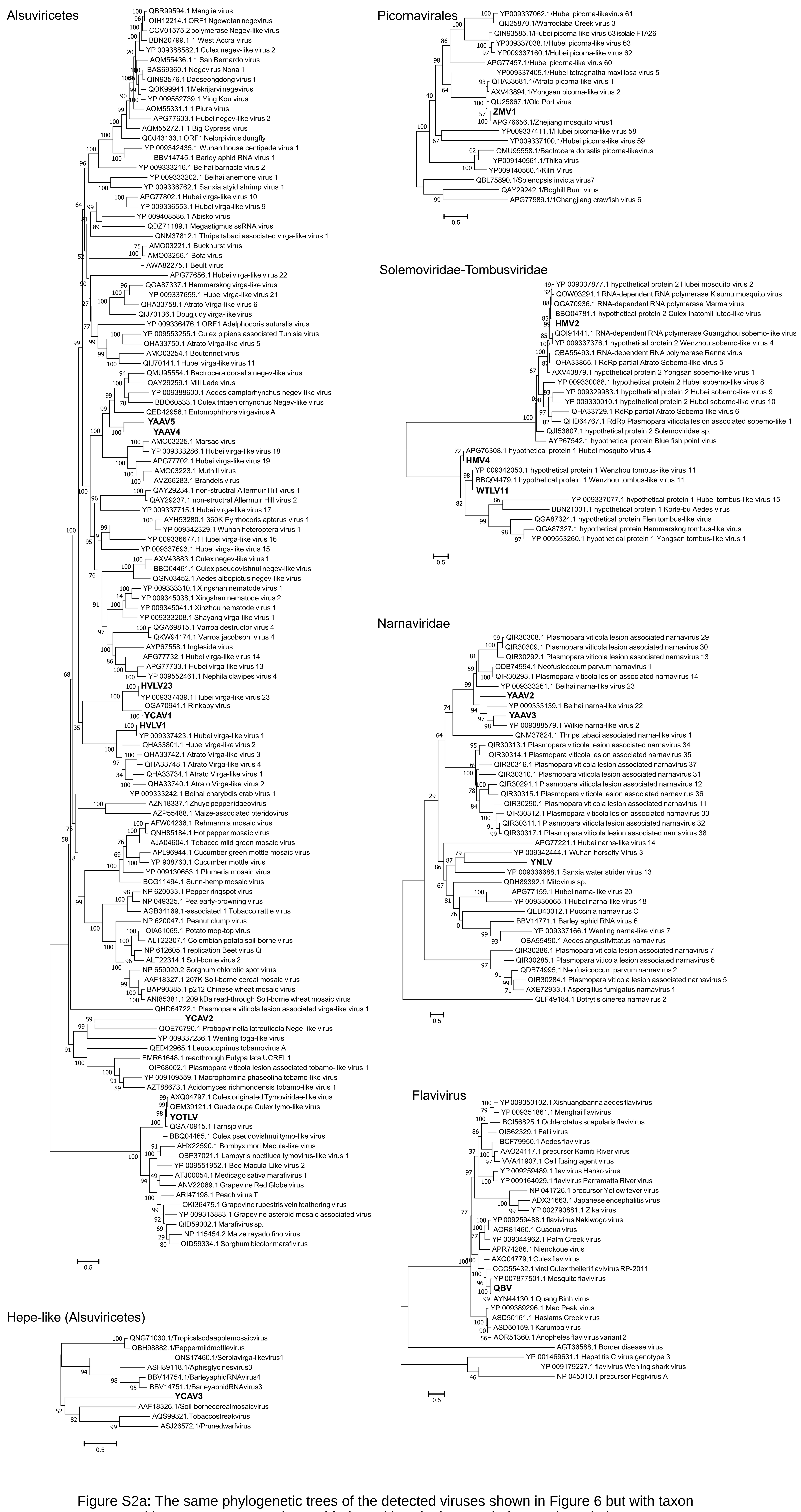

Figure S2a: The same phylogenetic trees of the detected viruses shown in Figure 6 but with taxon names and bootstrap support values added: Positive single-stranded RNA virus phylogeny trees

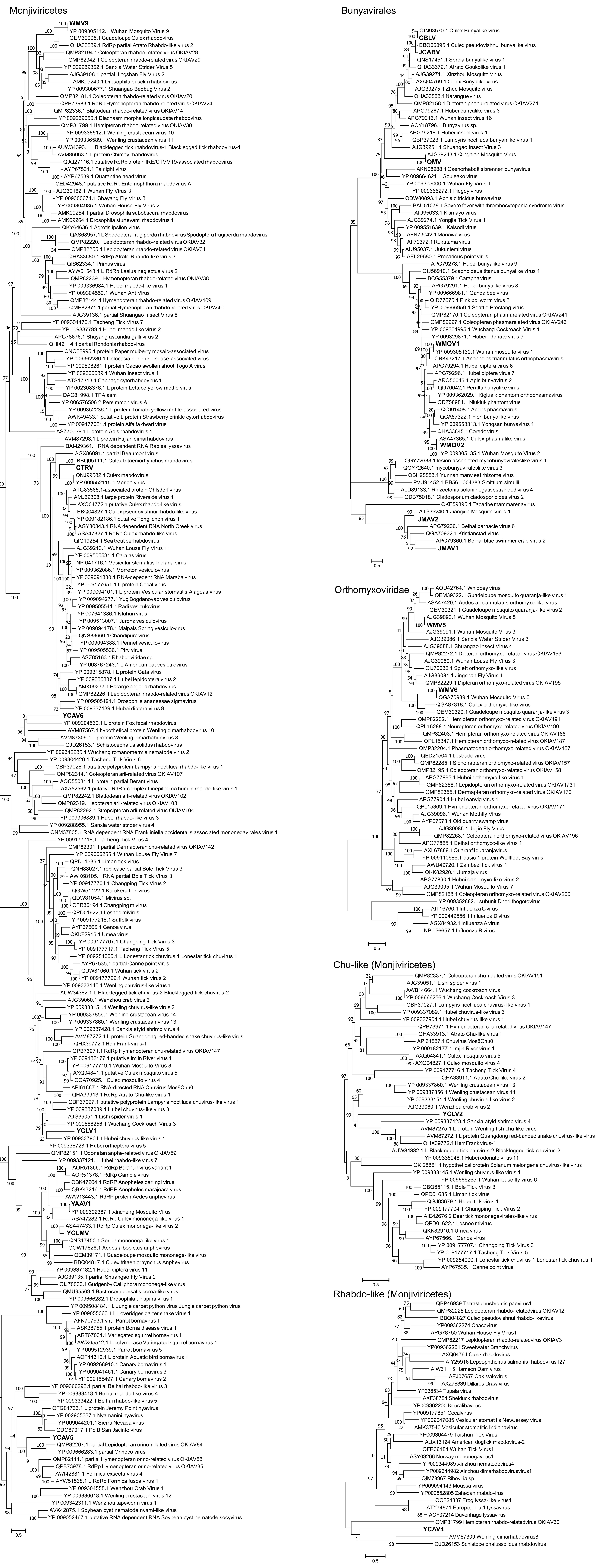

Figure S2b: The same phylogenetic trees of the detected viruses shown in Figure 6 but with taxon names and bootstrap support values added: negative single-stranded RNA virus phylogeny trees

Durnavirales

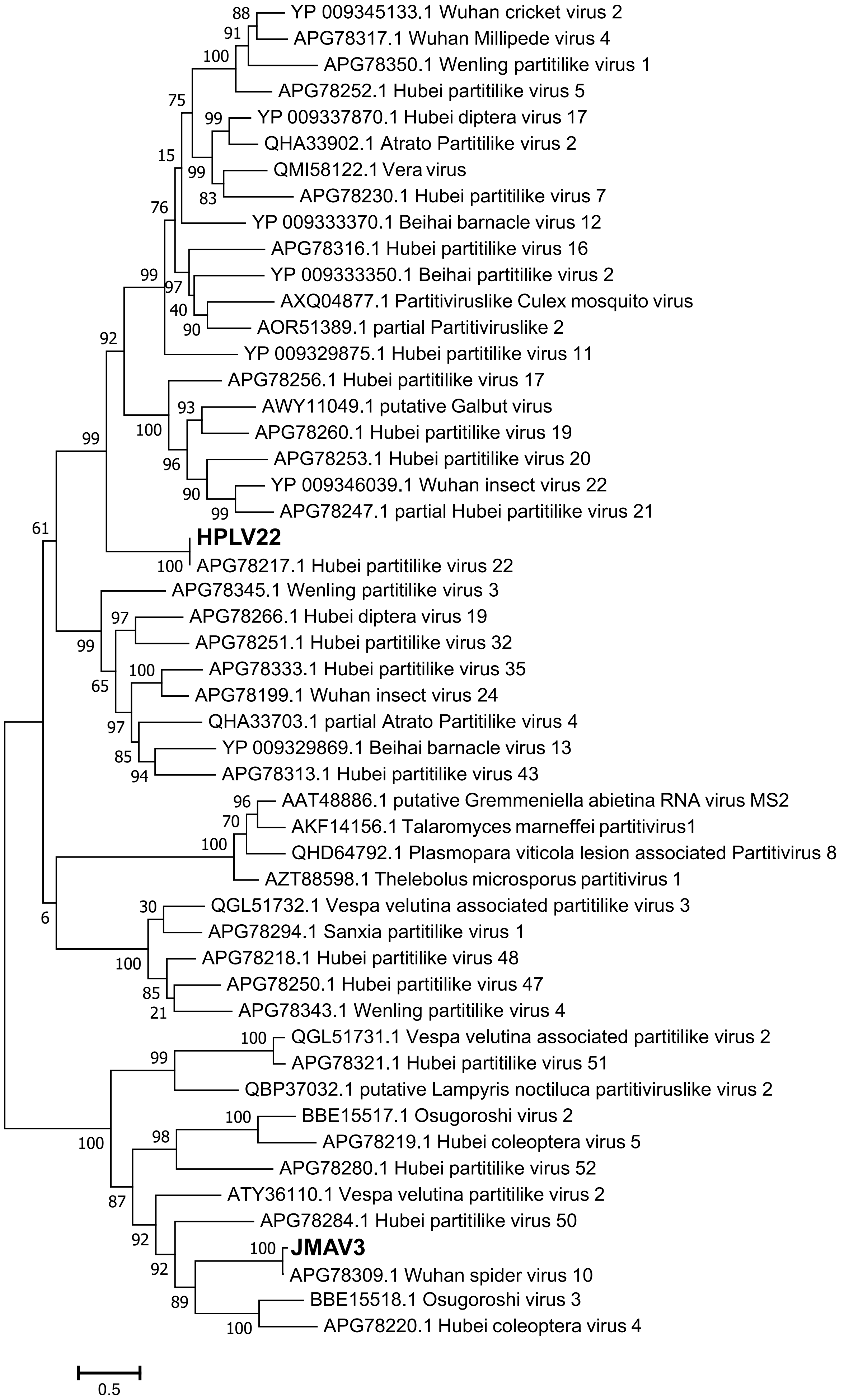

Ghabrivirales

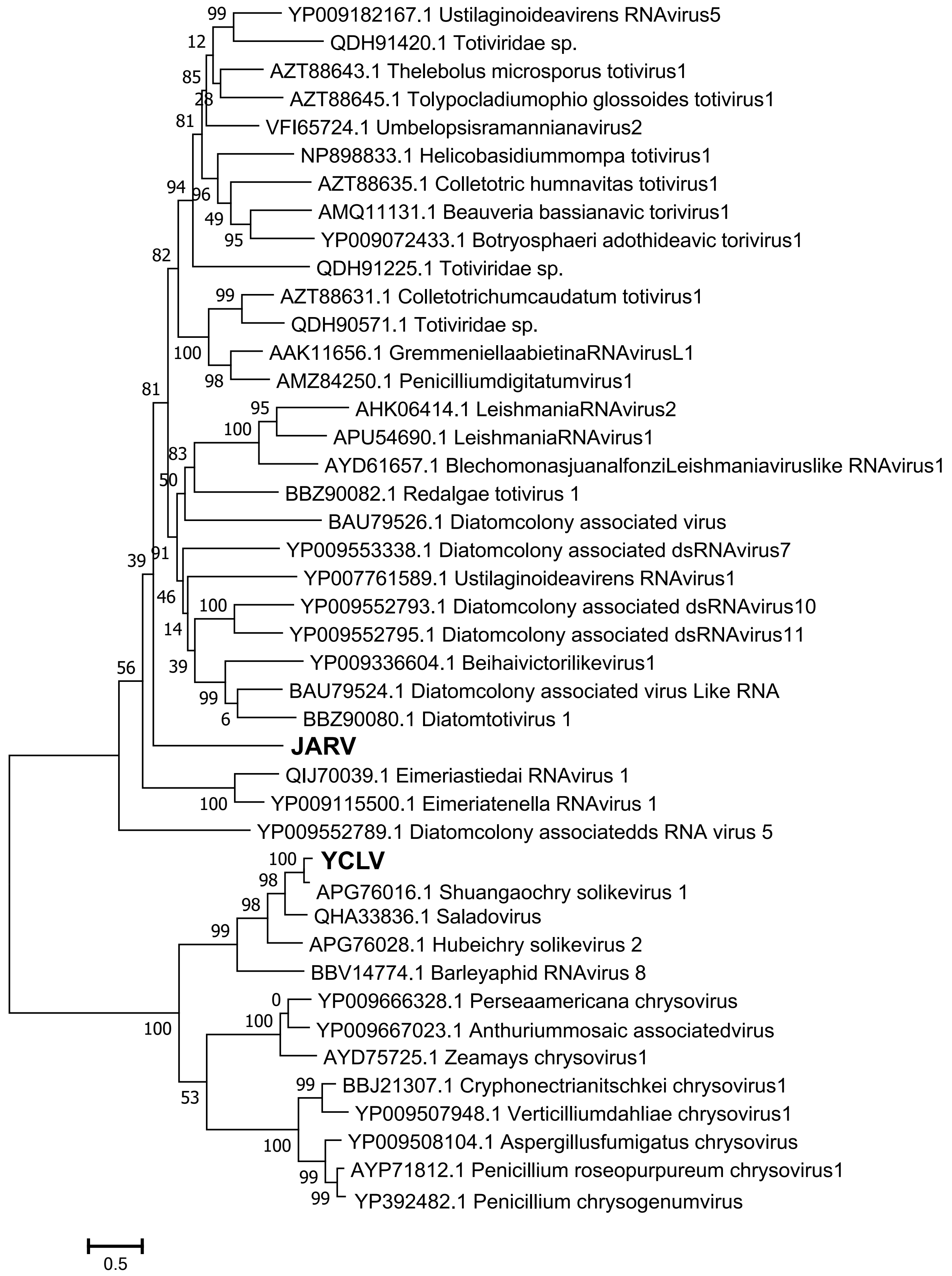

Reoviridae

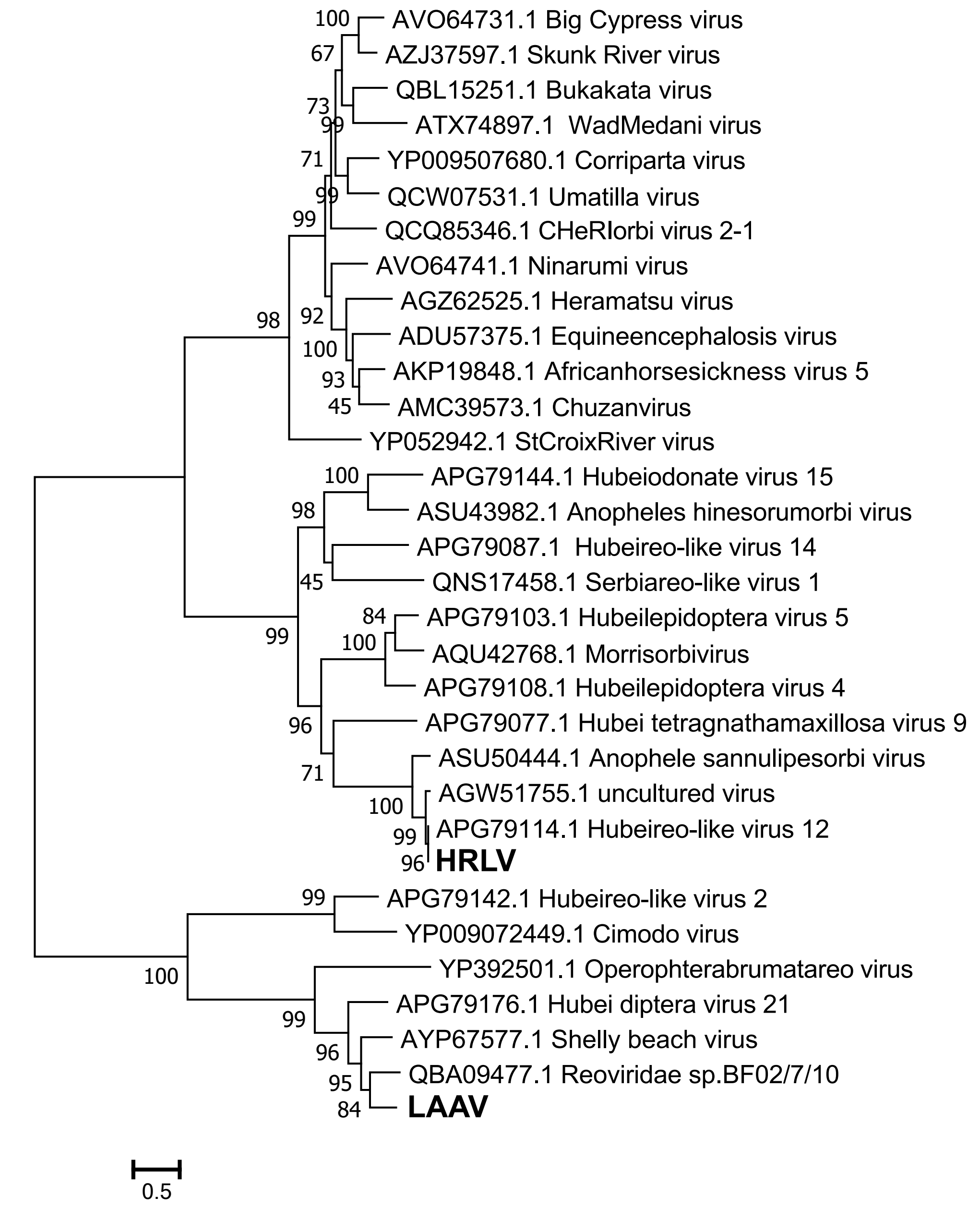

Figure S2c: The same phylogenetic trees of the detected viruses shown in Figure 6 but with taxon names and bootstrap support values added: double-stranded RNA virus phylogeny trees
